# Endovascular treatment of different types of iliac occlusions—Results from an observational study

**DOI:** 10.1371/journal.pone.0222893

**Published:** 2019-10-02

**Authors:** Vladimir Cvetic, Dragan Sagic, Igor Koncar, Vladimir Kovacevic, Oliver Radmili, Zelimir Antonic, Borivoje Lukic, Nikola Aleksic, Lazar Davidovic, Djordje Radak

**Affiliations:** 1 School of Medicine, Belgrade University, Belgrade, Serbia; 2 Clinic for Vascular and Endovascular Surgery, Clinical Center of Serbia, Belgrade, Serbia; 3 Institute for Cardiovascular Diseases “Dedinje”, Belgrade, Serbia; University Hospital Basel, SWITZERLAND

## Abstract

**Objective:**

The aim of this study was to evaluate the results of endovascular therapy on the treatment of different types of iliac occlusions.

**Materials and methods:**

A bi-center prospective, non-randomized study was conducted on 100 patients (mean age 59.14 ± 8.53; 64 men) who underwent endovascular treatment of iliac occlusive disease between January 2013 and November 2017. We evaluated baseline data, procedure, and follow-up results for the entire group, and according to Trans-Atlantic Inter-Society Consensus (TASC II) classification. The majority of patients (60%) were treated for severe claudication; 56 (56%) patients had TASC B occlusions, 28 patients TASC C, and 16 patients TASC D.

**Results:**

The mean length of the occluded segments was 61.41 ± 35.15 mm. Procedural complications developed in 6 patients (6%). Mean ankle-brachial pressure index increased from 0.40 ± 0.12 preoperatively to 0.82 ± 0.16 postoperatively. The mean follow-up was 33.18 ± 15.03 months. After 1 and 5 years, the primary patency rates were 98% and 75.1%, and the secondary patency rate was 97% respectively. Regarding occlusion complexity there were no statistical significant differences in primary patency rates (TASC B *vs*. C *vs*. D: p = 0.19). There were no statistically significant differences in primary patency rates between patients in different clinical stages, as well as between the type of stents, and location of the occlusion.

**Conclusion:**

In our study, endovascular treatment for iliac artery occlusions proved to be a safe and efficient approach with excellent primary and secondary patency rates regardless of the complexity of occlusions defined by TASC II classification. This study is aligned with the notion that in well selected patients, endovascular therapy can be the treatment of choice even in complex iliac lesions if performed by experienced endovascular interventionists in high volume centers.

## Introduction

During the last decade, the application of endovascular treatment as a therapeutic option for the aorto-iliac occlusive disease has continually increased, becoming the treatment of the first choice for many of the Trans-Atlantic Inter-Society Consensus document II (TASC II) categories [1,2]. The 2007 TASC II document stated an endovascular-first strategy up to type B lesions and surgery for low-risk patients with type C and D lesions, underscoring that the reasoning behind the decisions should be based on the comorbidities of the patient and the success rates of the operator [3]. European Society of Cardiology (ESC) guidelines from 2011 recommend endovascular treatment for all iliac TASC A-C lesions and TASC D lesions in patients with severe comorbidities, provided that the procedure is performed by an experienced team [4]. The likely risk of complications and lower patency of complex lesions are the reasons why the endovascular option in more complex lesions is not advised. The 2015 revision of TASC II document shares the same view, implying that the method of revascularization should be chosen depending on the competence of a vascular center and its experience, considering patient comorbidity and overall prognosis, supporting the endovascular-first approach in all iliac lesions in highly experienced centers [5]. Recent 2017 ESC Guidelines, in collaboration with the European Society for Vascular Surgery (ESVS), are recommending an endovascular-first strategy for aorto-iliac occlusive lesions if the procedure is performed by an experienced team and if it does not compromise subsequent surgical options [6]. These recent changes are based on expert opinions presented in the studies from high volume centers. They reflect both the increase of endovascular experience and technical advancements over the last two decades, resulting in a rising number of centers which opt for endovascular-first approach even in complex TASC C and D lesions [7,8]. The rationale for offering an endovascular-first option to the patient with complex lesion would be a low risk of complications and long-term patency, also it is important to notice that perioperative morbidity in surgically treated patients is substantial, and the time-period before return to normal activities is shorter in endovasculary treated patients.

The aim of this study was to evaluate and compare early and long-term results of the endovascular treatment among patients with different types of occlusions in the iliac segment.

## Materials and methods

### Patient population

**Fig 1:**
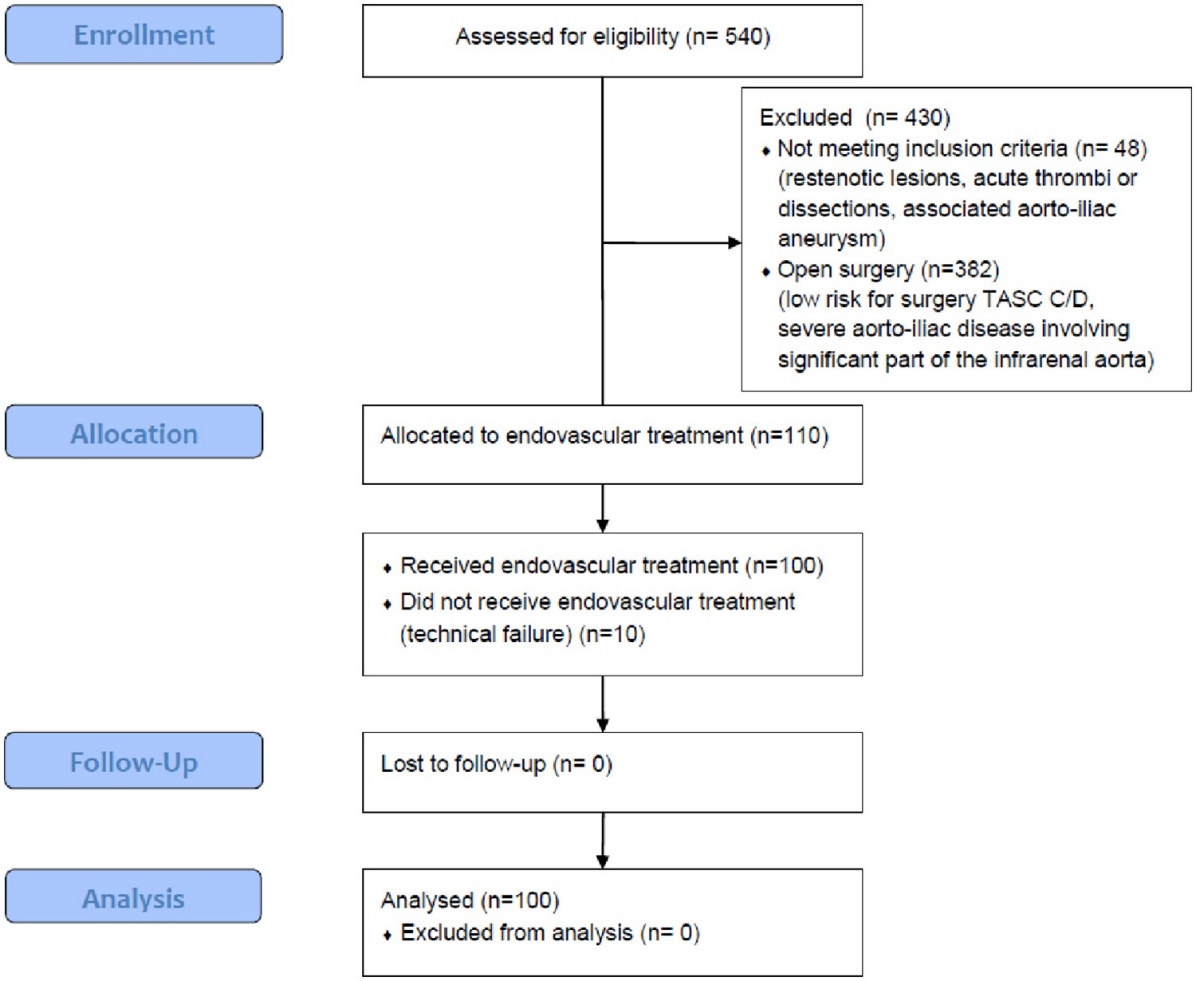
Study flowchart.

This is a prospective, non-randomized, bi-center cohort study conducted at two vascular centers in Belgrade, Serbia: the Clinic for Vascular and Endovascular Surgery of the Clinical Center of Serbia and the Institute for Cardiovascular Diseases “Dedinje”. Between January 2013 and November 2017 a total of 540 patients with TASC B, C and D occlusions in the aorto-iliac segment were considered for the study at these two centers. All patients with TASC B occlusions were included in the study. The conditions for including patients with TASC C and D occlusions were if their occlusive lesion was considered suitable for endovascular treatment and high risk for open surgery: older than 70 years, cardiac failure with ejection fraction lower than 40%, symptomatic coronary disease, and hostile abdomen. TASC C and D patients with severe aorto-iliac disease involving significant part of the infrarenal aorta and TASC C/D patients with low risk for surgery were treated by open surgery. Exclusion criteria were associated with aorto-iliac aneurysm, restenotic lesions, acute thrombi or dissections. Patients with an initial technical failure (inability to cross an occluded arterial segment) (10/110 (9.1%) patients) were excluded from the study and were treated by immediate surgical repair. Finally, 100 patients were included in the study (68 patients at the Clinic for Vascular and Endovascular Surgery of the Clinical Center of Serbia, and 32 patients at the Institute for Cardiovascular Diseases “Dedinje”; Fig 1).

All patients had evidence of chronic limb ischemia classified according to Rutherford (stage 3 or greater). The TASC II classification was used to define the characteristics of the lesions. The study protocol was approved by the local ethics committee (Ethics Committee Faculty of Medicine University of Belgrade, decision 29/XI-17), and all patients gave informed written consent for participation in the trial. Demographic, clinical, and procedural data and outcomes were obtained from institutional medical records and angiograms. The study was registered at clinicaltrials.gov, registration number NCT03824730.

### Technical details

The preoperative diagnostic assessment consisted of clinical examination, ankle-brachial pressure index (ABI) measurement, and duplex ultrasonography examinations to determine the need for treatment. All patients had computed tomography angiography (CTA) to evaluate the lesion characteristics. The endovascular procedures were performed in the angiography suite by four experienced endovascular interventionalists from two high-volume vascular centers. All interventionalists underwent similar endovascular training, and had comparable endovascular procedure volumes (>100 procedures per year) and complication rates (<5%) in the past five years. Under local anesthesia, initial percutaneous access was chosen by operator depending on anatomical and morphological occlusion characteristics. Heparin was administered in all cases, intra-arterially at a dose of 5000 IU after placement of a vascular sheath. Iliac lesion crossing was achieved through intraluminal or subintimal manner depending on the behavior of the lesion intraoperatively. Reentry devices were not used. Predilatation of the occlusion before stent deployment was performed at the discretion of the operator. Balloon-expandable (Omnilink Elite, Abbott Vascular, Santa Clara, CA, USA; Express, Boston Scientific, Galway, Ireland; Assurant Cobalt, Medtronic, Galway, Ireland) and self-expanding stents (EverFlex, Medtronic, Galway, Ireland; Epic, Boston Scientific, Galway, Ireland; Smart, Cordis, Santa Clara, CA, USA) were deployed in all lesions. Covered stents (E-ventus, Jotec, Hechingen, Germany) were used just in case of iliac artery rupture. Technical success was defined as residual stenosis <30% on final intraoperative arteriography [9]. Two endovascular interventionalists who were blinded to the treatment strategy rated the final angiography. Postprocedural medical treatment consisted of dual-antiplatelet therapy for 3 months, and then patients remained to aspirin only. Procedural and periprocedural data included approach, the procedure performed, type, number, diameter and length of stents.

### Follow-up protocol

All patients were followed up with clinical evaluation, ABI, and duplex ultrasonography examinations at 1, 6, and 12 months after the intervention, and then annually. The evaluation was also performed when new clinical symptoms developed. The follow-up was stopped in September 2018. Early results (procedural and <30 days) were analyzed for periprocedural complications (access site complications, distal embolization, perforation, surgical repair, myocardial infarction, stroke, death), primary patency (defined as uninterrupted patency with no procedure or intervention performed on or at the margin of the treated segment), and secondary patency (defined as restored patency through the previously treated segment). Primary patency loss was determined when ABI deterioration >20% was associated with duplex evidence of hemodynamic significant (>70%) restenosis or occlusion [9]. In the presence of clinical symptoms and flow-limiting lesions or thrombosis of the treated segment, patients underwent CTA before secondary interventions. Follow-up results were analyzed for primary and secondary patency rates.

### Statistical analysis

Sample size based on confidence interval (CI) for a proportion with the 95% CI (p = 0.14 and W = 0.06), in our study with 514 patients was found at least 72 endovascular patients to be sufficient for detection of significant difference of observed parameters [10,11]. For normal distribution data testing, the Kolmogorov-Smirnov and Shapiro-Wilk tests were used. Descriptive methods (frequencies, percent, mean, median, standard deviation /SD/ and range) were used to summarize the data. The statistical significance level was set at p<0.05 with Bonferroni correction for multiple testing at the same data set. For characteristics comparison among different risk subgroups Kruskal-Wallis, Wilcoxon rank sum, Pearson chi-square, and Fisher exact tests were used. Wilcoxon signed rank test with continuity correction was used for preoperative ABI and postoperative ABI comparison. Methods of Survival analysis were used for primary patency rates, and secondary patency rates (Kaplan-Meier product-limit method; cumulative percentage with corresponding 95% CI and log-rank test). The statistical analysis was done with the program R (version 3.3.2 (2016-10-31)—"Sincere Pumpkin Patch"; Copyright (C) 2016 The R Foundation for Statistical Computing; Platform: x86_64-w64-mingw32/x64 (64-bit); downloaded: January 21, 2017).

## Results

### Patient characteristics

In total, 100 patients from 2 vascular centers underwent endovascular procedures for iliac occlusions. Patient characteristics are listed in Table 1 for the whole group and according to the TASC II classification.

**Table 1:** Demographic/Clinical characteristics: Whole group and according to TASC II classification.

Characteristics	Total	TASC II classification			
		TASC B	TASC C	TASC D	P value
**Gender**					
- Male	64 (64%)	34 (60.71%)	20 (71.43%)	10 (62.50%)	0.62
- Female	36 (36%)	22 (39.29%)	8 (28.57%)	6 (37.50%)	
**Age (years)**					
- Mean ± SD	59.14 ± 8.53	58.45 ±8.43	59.93 ± 8.38	60.19 ± 9.44	0.54
**Risk Factors**					
- Smoking	70 (70%)	40 (71.43%)	21 (75%)	9 (56.25%)	0.40
- Hypertension	63 (63%)	39 (69.64%)	16 (57.14%)	8 (50%)	0.27
- Hyperlipidemia	37 (37%)	26 (46.43%)	9 (32.14%)	2 (12.50%)	**0.04**
- Diabetes mellitus	30 (30%)	15 (26.79%)	10 (35.71%)	5 (31.25%)	0.70
- (ex) smoker	18 (18%)	8 (14.29%)	7 (25%)	3 (18.75%)	0.49
**Comorbidities**					
- Coronary artery disease	39 (39%)	17 (30.36%)	16 (57.14%)	6 (37.50%)	0.06
- Carotid artery stenosis	18 (18%)	11 (19.64%)	4 (14.29%)	3 (18.75%)	0.88
- ESRD^a^	6 (6%)	2 (3.57%)	1 (3.57%)	3 (18.75%)	0.09
- Cerebral vascular disease	9 (9%)	5 (8.93%)	2 (7.14%)	2 (12.50%)	0.79
- COPD^b^	14 (14%)	6 (10.71%)	4 (14.29%)	4 (25%)	0.31
- Atrial fibrillation	7 (7%)	2 (3.57%)	3 (10.71%)	2 (12.50%)	0.28
**Rutherford Classification**					
- Class 3	60 (60%)	38 (67.86%)	14 (50%)	8 (50%)	0.50
- Class 4	27 (27%)	13 (23.21%)	9 (32.14%)	5 (31.25%)	
- Class 5	9 (9%)	4 (7.14%)	3 (10.71%)	2 (12.50%)	
- Class 6	4 (4%)	1 (1.79%)	2 (7.14%)	1 (6.25%)	
**Total patients**	**100 (100%)**	**56 (100%)**	**28 (100%)**	**16 (100%)**	**-**

Abbreviations:

^a^ end-stage renal disease;

^b^ chronic obstructive pulmonary disease.

The group consisted of patients with a mean age of 58.5 years, predominantly males (64%, Table 1). The most common risk factors were a history or present status of smoking, followed by hypertension, and hyperlipidemia. Cardiovascular comorbidities were found in the majority of patients: most commonly coronary artery disease (39%) and carotid artery stenosis (18%, Table 1). According to Rutherford classification, the majority of patients (60%) were treated for severe claudication (Rutherford category 3), while 40% for critical limb ischemia (CLI) (Rutherford category 4, 5, 6; Table 1).

Results of the analysis showed that most of the basic characteristics (demographic/clinical) according to the TASC categories (B, C, and D) were homogeneous except hyperlipidemia which is the more common risk factor in patients with TASC B (46.4%; p = 0.04; Table 1).

### Lesion and procedural characteristics

Lesion and procedural characteristics are listed in Table 2.

**Table 2:** Lesion and procedural characteristics: Whole group and according to TASC II classification.

Characteristics	Total	TASC II classification			
		TASC B	TASC C	TASC D	P value
**Location**					
- CIA^a^	52 (52%)	49 (87.50%)	3 (10.71%)	0 (0%)	**4.35x10**^**-21**^
- EIA^b^	29 (29%)	7 (12.50%)	21 (75%)	1 (6.25%)	
- CIA + EIA	17 (17%)	0 (0%)	4 (14.29%)	13 (81.25%)	
- AORTA + CIA	2 (2%)	0 (0%)	0 (0%)	2 (12.50%)	
**Location (side)**					
- Left	41 (41%)	22 (39.29%)	14(50%)	5 (31.25%)	0.09
- Right	56 (56%)	34 (60.71%)	13 (46.43%)	9 (56.25%)	
- Both	3 (3%)	0 (0%)	1(3.57%)	2 (12.50%)	
**Initial Access**					
- Ipsilateral	34 (34%)	30 (53.57%)	2 (7.14%)	2 (12.50%)	**4.97x10**^**-8**^
- Contralateral	29 (29%)	6 (10.71%)	19 (67.86%)	4 (25%)	
- Both femoral	31 (31%)	18 (32.14%)	5 (17.86%)	8 (50%)	
- Left brachial	4 (4%)	1 (1.79%)	1 (3.57%)	2 (12.50%)	
- Brachial + Femoral	2 (2%)	1 (1.79%)	1 (3.57%)	0 (0%)	
**Occlusion length**					
- Mean ± SD	61.41 ± 35.15	39.02 ± 11.77	71 ± 20.8	123 ± 27.92	**1.71x10**^**-14**^
**Stent type**					
- Self-expanding	29 (29%)	9 (16.07%)	14 (50%)	6 (37.5%)	
- Balloon-expandable	54 (54%)	45 (80.36%)	7 (25%)	2 (12.50%)	**3.37x10**^**-9**^
- Both	17 (17%)	2 (3.57%)	7 (25%)	8 (50%)	
**Stent total length**					
- Mean ± SD	92.47 ± 49.21	64.79 ± 27.09	102.3 ± 35.33	172.20 ± 34.96	**2.83x10**^**-12**^
**Stent diameter**					
- Mean ± SD	8.47 ± 1.27	8.73 ± 1.1	7.61 ± 1.17	9.06 ± 1.29	**1.18x10**^**-4**^
**Number of stents** **(mean ± SD)**	1.84 ± 0.99	1.46 ± 0.76	1.82 ± 0.82	3.19 ± 0.83	**5.14x10**^**-8**^
**Number of stents**					
- One stent	48 (48%)	37 (66.07%)	11 (39.29%)	0 (0%)	**1.30x10**^**-7**^
- Two stents	30 (30%)	14 (25%)	12 (42.86%)	4 (25%)	
- Three stents	12 (12%)	3 (5.36%)	4 (14.29%)	5 (31.25%)	
- Four stents	10 (10%)	2 (3.57%)	1 (3.57%)	7 (43.75%)	
**Procedural complications**	6 (6%)	2 (3.57%)	1 (3.57%)	3 (18.75%)	0.09
**Pre ABI**^c^					
- Mean ± SD	0.40 ± 0.12	0.41 ± 0.13	0.4 ± 0.11	0.35 ± 0.08	**0.03**
**Post ABI**^d^					
- Mean ± SD)	0.82 ± 0.16	0.84 ± 0.16	0.77 ± 0.19	0.85 ± 0.14	0.18
**Pre ABI vs. post ABI**	**2.20x10**^**-16**^	**2.20x10**^**-16**^	**2.85x10**^**-9**^	**1.67x10**^**-6**^	-
**Total patients**	**100 (100%)**	**56 (100%)**	**28 (100%)**	**16 (100%)**	**-**

Abbreviations:

^a^ common iliac artery;

^b^ external iliac artery;

^c^ preoperatively ankle brachial pressure index;

^d^ postoperatively ankle brachial pressure index.

The most frequent location of iliac artery occlusions was common iliac artery (CIA) in TASC B lesions (85.7%), external iliac artery (EIA) in TASC C (75%), and CIA+EIA in TASC D group (81.2%; p = 4.35x10^-21^; Table 2). The distal part of the aorta and both CIA were occluded in 2 patients (12.5%) in the TASC D group.

The most common initial access was: ipsilateral in TASC B occlusions (53.6%), contralateral in TASC C lesions (42.9%) and both femoral in TASC D lesions (50%; p = 4.97x10^-8^; Table 2). The left brachial, and simultaneous left brachial and femoral approach were used for 6 patients (6%).

In the entire study, the mean length of occluded arterial segments was 61.41 ± 35.15 mm. As expected, occlusion length was higher in TASC D lesions, then in TASC C, and the shortest in TASC B occlusions (mean values were 123, 71, and 39.02 respectively; p = 1.71x10^-14^; Table 2).

Predilatation was performed in 93 patients. Balloon-expandable stents were the most common in TASC B lesions (80.4%) compared with TASC C (self-expanding, 50%), and TASC D (both stents, 50%; p = 3.37x10^-9^; Table 2).

The number of implanted stents was higher in TASC D lesions compared with TASC B and C lesions (mean values were 3.19, 1.82 and 1.46 respectively; p = 5.14x10^-8^; Table 2). In other words, most of the patients with TASC D occlusions were treated with 4 stents (43.7%) which is significantly higher than TASC C (commonly 2 stents, 42.9%), and TASC B (commonly 1 stent, 66.1%; p = 1.30x10^-7^; Table 2).

Following procedure, the ABI significantly increased in all the patients (pre ABI vs. post ABI mean values were 0.4 vs. 0.82; p = 2.20x10^-16^; Table 2) as same as in each TASC category (pre ABI vs. post ABI mean values in TASC B, C and D were 0.41 vs. 0.84 (p = 2.20x10^-16^); 0.4 vs. 0.77 (p = 2.85x10^-9^); 0.35 vs. 0.85 (p = 1.67x10^-6^); respectively, for subgroups TASC B, C and D; Table 2), so there was no statistical difference in post ABI values between TASC categories (Table 2).

Periprocedural complications developed in 6 patients (6%). Two iliac ruptures occurred, one in TASC C occlusion, another in TASC D lesion, and both were successfully treated with balloon expandable covered stents. One patient developed groin hematoma that did not require further active care. There was a case of a retroperitoneal hematoma, without the need for drainage. One distal embolization was corrected by embolectomy using a Fogarty catheter. Patient with arterial pseudoaneurysm was successfully treated by ultrasound-guided manual compression. There was no statistically significant difference between groups (TASC B, C, D) regarding periprocedural complications (p = 0.09).

### Follow-up results

The median follow-up period was 33.18 ± 15.03 months (range, 10–62 months), and none of the patients was lost to follow-up. 24 patients were followed for 48–62 months, 20 patients for 36–48 months, and for 23 patients follow-up was done between 24–36 months. Most patients (30) had a last control between 12–24 months, and only 3 patients were followed for 10 months. Two patients died (cumulative mortality: 2%) during follow-up: the cause of death was cardiac in one patient, and the other died due to pancreatic cancer.

In two patients, in-stent restenosis (>70%) was detected at 26 and 33 months: one in TASC B lesion and one in TASC D lesion. These cases were successfully corrected–one with the drug coated balloon angioplasty, another with new stent placement.

Eight lesions occluded during the follow-up period, at 1, 9, 13, 20, 28, 33, and 53 months. Five patients were treated with secondary endovascular intervention and three patients with open surgery. One patient suffered iliac thrombosis 3 months after secondary endovascular intervention and underwent to aortobifemoral bypass. No major amputation was recorded.

**Fig 2:**
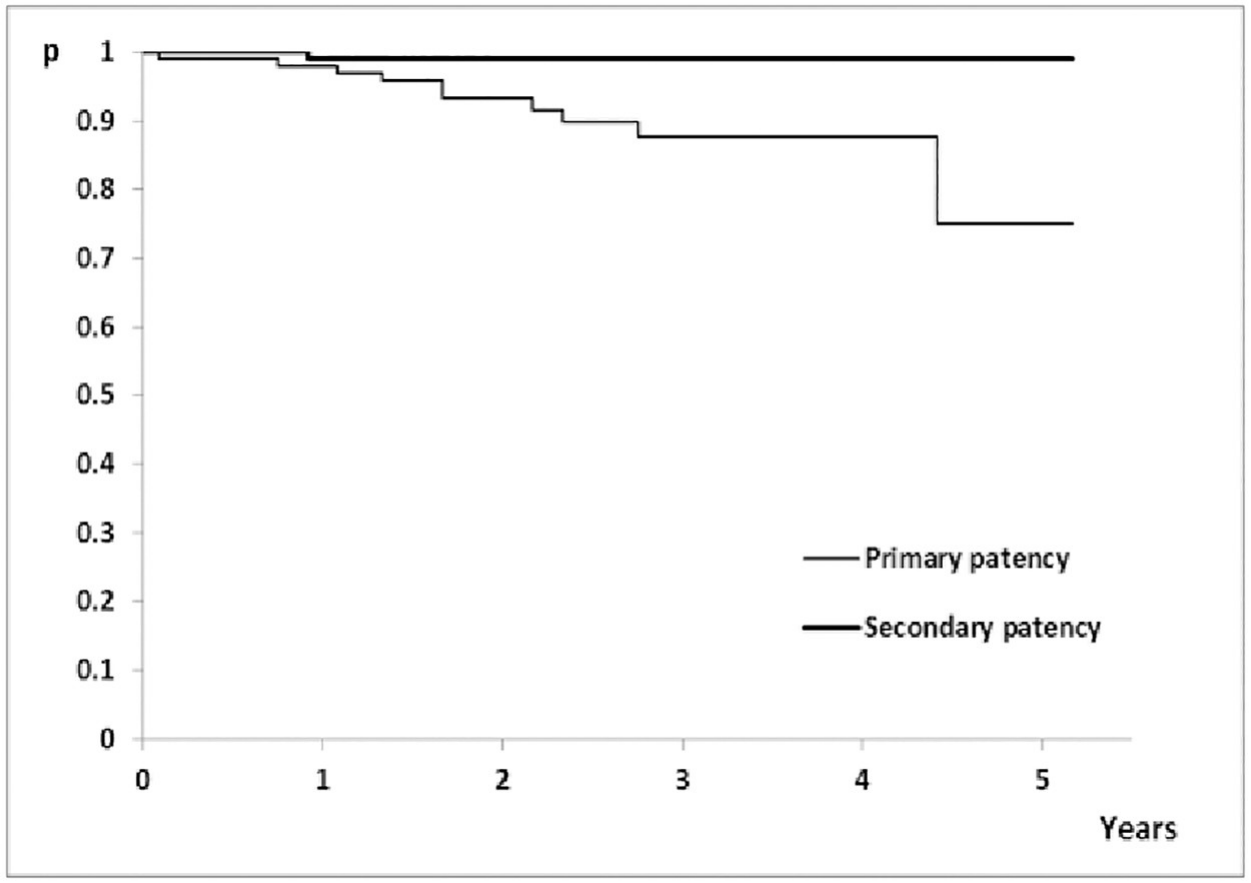
Kaplan-Meier primary and secondary patency rates for the whole group.

For the whole study cohort, primary patency was estimated to be 98% (95% CI: 95.3–100%) after 1 year, after 3 years 87.7% (95% CI: 80.2–95.9%), and still 75.1% (95% CI: 54.8–100%) after 5 years, and secondary patency was 97% respectively. (Fig 2).

**Fig 3:**
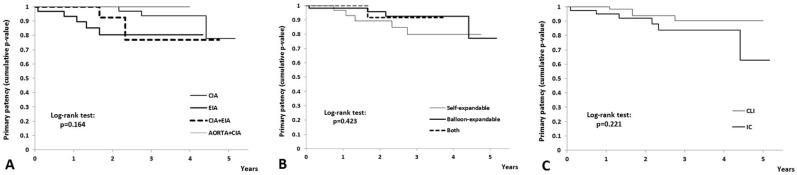
Kaplan-Meier primary patency rates. **(A)** For aorto-iliac segment subjected to endovascular treatment. (B) For stent type. (C) For patients with CLI and IC.

According to the location of aorto-iliac occlusion subjected to endovascular treatment, there was no statistically significant difference regarding the primary patency rates (CIA (52 patients) *vs*. EIA (29 patients) *vs*. CIA + EIA (17 patients) *vs*. Aorta + AIC (2 patients): p = 0.16; Fig 3).

The type of stent had no statistical significant impact on primary patency rates (self-expanding (29 patients) *vs*. balloon-expandable (54 patients) *vs*. both (17 patients), p = 0.42; Fig 3).

The estimated primary patency rates for CLI patients (60 patients) were 95% after 1 year, and 62.8%, after 5 years, whereas for intermittent claudication (IC) patients (40 patients) were 100% after 1 year, and 90.4% after 5 years (Fig 3). There was no statistically significant difference between CLI patients as compared to IC patients, regarding the primary patency rates (p = 0.22).

**Fig 4:**
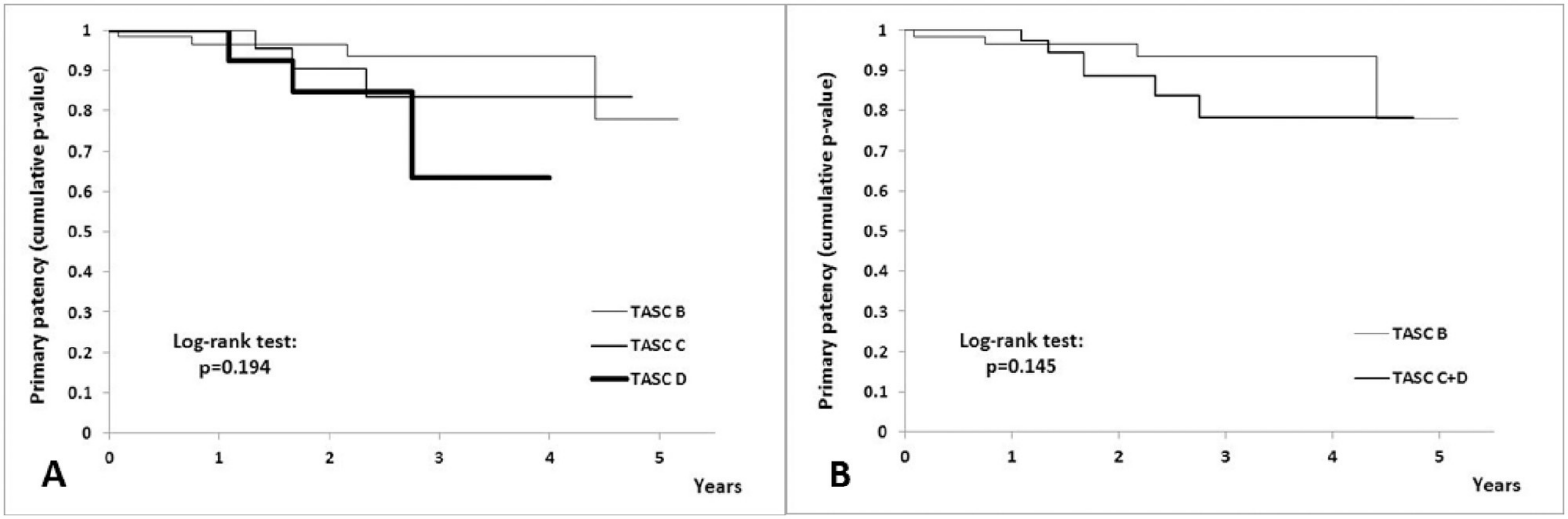
Kaplan-Meier primary patency rates for TASC II classification. (A) TASC B *vs*. TASC C *vs*. TASC D. (B) TASC B *vs*. TASC C/D.

Stratifying patients according to TASC II classification, we found no statistically significant differences between B, C and D types of occlusions concerning primary patency rates (TASC B (56 patients) *vs*. C (28 patients) *vs*. D (16 patients); p = 0.19 Fig 4).

Moreover, there were no statistically significant differences between TASC B (56 patients) and more complex TASC C/D (44 patients) lesions (p = 0.14; Fig 4).

## Discussion

In this study, we have analyzed our early and long-term results of endovascular treatment of iliac occlusions, also comparing patients treated for iliac occlusions according to the TASC categories (TASC B, C, D).

Since the risk of perioperative complications during open surgery is considerable, and the materials and techniques of endovascular surgery have greatly improved, the endovascular approach has become the first-choice therapeutic option for almost all patients with aorto-iliac occlusive disease [9,11–14]. In our two centers majority of patients with aorto-iliac occlusive disease were with TASC C/D lesions. Most complex TASC C/D patients with infrarenal aortic occlusions and with severe aorto-iliac occlusive disease involving significant part of the aorta were selected for open surgery. Remaining TASC C/D patients with distal aortic occlusions and long iliac occlusions that were suitable were endovasculary treated.

In all cases, CTA was performed before the procedure, which enabled us to evaluate the quality of the lesion and accurately measure the lengths and diameters, to be able to decide upon the adequate access and select the correct materials.

All procedures were performed percutaneously. The ipsilateral femoral approach was mostly used for patients with TASC B lesions (53.6%). TASC C occlusions more frequently underwent contralateral approach (67.9%). Both femoral accesses were necessary for half of the patients with TASC D occlusions. *Nyman* et al. [15] report that the low success rate in the reconstruction of iliac occlusions is because of the lack of attempts to use a brachial access site. *Ahn* et al. [16] treated almost all complex TASC D lesions (77.7%) with left brachial access. In our study, a left brachial and simultaneous left brachial and femoral approach was reserved for reconstruction of the aortic bifurcation or complex ostial occlusions.

In our series, routine stenting was used in all cases, which is congruent with the *AbuRahma* et al. study [17]. Stenting with predilatation was preferred in the majority of our patients. Also, *Yuan* et al. [18] in their study preferred to perform predilatation, which could reduce the length of the stents. Conversely, in several other studies, primary stenting has been preferred of extensive iliac artery occlusions because stent placements without predilatation reduce the risk of artery rupture, and decrease the risk of distal embolism [19–20]. In our cohort, there was only one embolic event, and 2 iliac artery ruptures that were caused by predilatation. All of these events were successfully treated.

All commercially available types of stents were used: we preferred balloon-expandable stents in patients with calcified, ostial lesions, whereas self-expanding stents were implanted for tortuous arteries [14]. Both stents were used in long-segment lesions involving heavily calcified common iliac arteries. According to *Müller* et al. [21], the type of stent may influence the development of restenosis. They noticed a clear trend toward a lower estimated probability for the development of restenosis for balloon- as compared to self-expanding stents. In our study, there was no difference regarding primary patency for the type of stents (self-expanding *vs*. balloon-expandable *vs*. both).

Periprocedural and early results were satisfactory, with significant hemodynamic improvement. Also, the periprocedural complications were rare, involving primarily minor vascular access site complications (6%), which could be treated conservatively or minimal invasively, thus avoiding the need for surgery. Complication rates reported in other studies ranged between 10% and 24% [20,22–23]. *Suzuki* et al. [22] analyzed 2096 patients with aorto-iliac disease (stenosis and occlusions) from the Japanese multicenter registry and showed that complications were more frequent in the TASC D group (70.6% occlusions). In our study, all patients were with chronic total occlusions. In the most complex TASC D group complications rate was higher, but there was no statistically significant difference between TASC II categories (TASC B *vs*. C *vs*. D: 3.6% vs. 3.6% vs. 18.8%, p = 0.09).

Most patients in our study suffered from severe intermittent claudication, common in most published series–the percentage of claudicants being between 50% and 100% [14]. Within our study, stage of limb ischemia did not have a statistically significant effect on the outcomes of endovascular treatment of iliac occlusions, but we noticed nonsignificant trend toward a lower patency rate in patients with CLI. The estimated primary patency was 90.4% after 5 years in patients with severe claudication, and 62.8% in patients with CLI. *Ozkan* et al. [20] reported that the primary patency rate was higher in patients with intermittent claudication than in patients with CLI. However, *Soga* et al. [24], who analyzed 2147 stented iliac arteries after 5 years, found no statistical difference in the primary patency of claudication, and CLI group, which confirms our results. It is worthy of noting that our patients did not differ in their comorbidity rates regardless of the type of the lesion, which might be important detail advising us to always observe the patients in general, and not only the type of the lesion.

In our study, there were no statistical significant difference according location of aorto-iliac occlusion (CIA vs. EIA *vs*. CIA + EIA *vs*. aorta + CIA, p = 0.16). Similarly, *Soares* et al. [25] compared the outcomes between CIA and EIA *vs*. CIA, and they found that the primary patency rates were similar between the patient’s groups. In contrast, *Müller* et al. [21] reported that the estimated probability of freedom from occlusion was significantly lower for interventions within the common iliac artery as compared to the external iliac artery, claiming that the vessel and stent diameter were the key parameters concerning the development of restenosis.

In their systematic review, *Jongkind* et al. [1] involved a total of 1711 patients with the extensive aorto-iliac disease (TASC C/D lesions) who were treated with endovascular treatment. At 4 and 5 years, primary patency rates ranged from 60 to 86%. In our study, the primary patency rates at 5 years were 75.1% for the entire study population enrolled, and 78.2% for complex type C/D lesions. The long-term patency rate in our endovascular study after 5 years was comparable to recent work for open surgery of *Indes* et al. [26].

There were no differences in patency between different TASC II types of occlusions when the lesions were stratified and analyzed accordingly. Our study agreed with the results of several earlier studies indicating no differences in long-term patency between different TASC II types of occlusions [10,27–28]. This investigation represents the clinical practice of two high-volume vascular centers and suggests that more complex occlusions type C/D in well selected patients can be managed using endovascular approaches with excellent long-term success and low complication rates.

### Limitations

Several limiting aspects of this study should be considered before evaluating the results. First at all, this study included only endovascular procedures, so a comparison with surgical procedures will be important in the further development of aorto-iliac occlusive treatment. Procedural failures were not included in the analysis. Although the number of these patients was 9.1%, one should be aware that not all lesions and patients are feasible for endovascular repair. Also, the procedures reviewed in this study only included balloon angioplasty and stent deployment, as debulking devices, and reentry devices were unavailable to us, due to cost issues. This study included exactly 100 patients and certain parameters especially when stratifying the patients in groups may not have reached any statistically significant due to the limited number of patients in each group. Besides that, follow up period (mean (SD) were 33 (15) months) maybe had influence on long-term primary patency.

## Conclusions

In our prospective, non-randomized study, endovascular treatment for iliac artery occlusions proved to be a safe and efficient approach with excellent primary and secondary patency rates regardless of the complexity of occlusions defined by TASC II classification. This study is aligned with the notion that in well selected patients, endovascular therapy can be the treatment of choice even in complex iliac lesions if performed by experienced endovascular interventionists in high volume centers.

## Supporting information

S1 File Study datasets. (XLS)

S2 File Informations for participants in written form. (DOC)

## References

[1] Jongkind V, Akkersdijk GJ, Yeung KK, Wisselink W. A systematic review of endovascular treatment of extensive aortoiliac occlusive disease. J Vasc Surg 2010; 52:1376–83. 10.1016/j.jvs.2010.04.080 20598474

[2] Ye W, Liu CW, Ricco JB, Mani K, Zeng R, Jiang J. Early and late outcomes of percutaneous treatment of TransAtlantic Inter-Society Consensus class C and D aorto-iliac lesions. J Vasc Surg 2011; 53:1728–37. 10.1016/j.jvs.2011.02.005 21609804

[3] Norgren L, Hiatt WR, Dormandy JA, Nehler MR, Harris KA, Fowkes FG. Inter-Society Consensus for the Management of Peripheral Arterial Disease (TASC II). J Vasc Surg 2007; 45(suppl S): S5–S67. 17223489 10.1016/j.jvs.2006.12.037

[4] European Stroke Organisation, Tendera M, Aboyans V, Bartelink L, Baumgartner I, Clément D, et al ESC Guidelines on the diagnosis and treatment of peripheral artery diseases: document covering atherosclerotic disease of extracranial carotid and vertebral, mesenteric, renal, upper and lower extremity arteries: the Task Force on the Diagnosis and Treatment of Peripheral Artery Disease of the European Society of Cardiology (ESC). Eur Heart J 2011; 32(22):2851–2906. 10.1093/eurheartj/ehr211 21873417

[5] TASC Steering Committee, Jaff MR, White CJ, Hiatt WR, Fowkes GR, Dormandy J, et al An update on methods for revascularization and expansion of the TASC lesion classification to include below-the-knee arteries: a supplement to the Inter-Society Consensus for the Management of Peripheral Arterial Disease (TASC II): The TASC Steering Committee. Ann Vasc Dis 2015; 8(4):343–357. 10.3400/avd.tasc.15-01000 26730266 PMC4691515

[6] Aboyans V, Ricco JB, Bartelink MEL, Björck M, Brodmann M, Cohnert T, et al 2017 ESC Guidelines on the Diagnosis and Treatment of Peripheral Arterial Diseases, in collaboration with the European Society for Vascular Surgery (ESVS): Document covering atherosclerotic disease of extracranial carotid and vertebral, mesenteric, renal, upper and lower extremity arteriesEndorsed by: the European Stroke Organization (ESO)The Task Force for the Diagnosis and Treatment of Peripheral Arterial Diseases of the European Society of Cardiology (ESC) and of the European Society for Vascular Surgery (ESVS). Eur Heart J 2018; 39:763–816. 10.1093/eurheartj/ehx095 28886620

[7] Sixt S, Alawied AK, Rastan A, Schwarzwälder U, Kleim M, Noory E, et al Acute and long-term outcome of endovascular therapy for aortoiliac occlusive lesions stratified according to the TASC classification: a single-center experience. J Endovasc Ther 2008; 15(4):408–416. 10.1583/08-2359.1 18729553

[8] Bosiers M, Deloose K, Callaert J, Maene L, Beelen R, Keirse K, et al BRAVISSIMO: 12-month results from a large scale prospective trial. J Cardiovasc Surg (Torino) 2013; 54(2):235–253. 23558659

[9] Rutherford RB, Baker JD, Ernst C, Johnston KW, Porter JM, Ahn S, et al Recommended standards for reports dealing with lower extremity ischemia: revised version. J Vasc Surg 1997; 26: 517–38. 10.1016/s0741-5214(97)70045-4 9308598

[10] Leville CD, KAshyap VS, Clair DG, Bena JF, Lyden SP, Greenberg RK, et al Endovascular management of iliac artery occlusions: extending treatment to Trans Atlantic Inter-Society Consensus class C and D patients. J Vasc Surg 2006; 33:32–9. 10.1016/j.jvs.2005.09.034 16414384

[11] Hulley SB, Cummings SR, Browner WS, Grady D, Newman TB. Designing clinical research: an epidemiologic approach. 4^th^ ed Philadelphia, PA: Lippincott Williams & Wilkins; 2013 Appendix 6E, page 81.

[12] De Vries SO, Hunink MGM. Results of aortic bifurcation grafts for aorto-iliac occlusive disease: a meta-analysis. J Vasc Surg 1997; 26:558–69. 10.1016/s0741-5214(97)70053-3 9357455

[13] Timaran CH, Prault TL, Stevens SL, Freeman MB, Goldman MH. Iliac artery stenting versus surgical reconstruction for TASC type B and type C iliac lesions. J Vasc Surg 2003; 38:272–8. 12891108 10.1016/s0741-5214(03)00411-7

[14] Tsetis D, Uberoi R. Quality improvement guidelines for endovascular treatment of iliac artery occlusive disease. Cardiovasc Intervent Radiol 2008;31:238–45. 10.1007/s00270-007-9095-5 18034277

[15] Nyman U, Uher P, Lindh M, LAindblad B, Ivancev K. Primary stenting in infrarenal aortic occlusive disease. Cardiovasc Intervent Radiol 2000; 23:97–108. 10.1007/s002709910021 10795833

[16] Ahn S, Park KM, Kim YK, Kim JI, Moon IS, Hong KC, et al Outcomes of endovascular treatment for TASC C and D aorto-iliac lesions. Asian J Surg 2017; 40(3):215–20. 10.1016/j.asjsur.2015.11.006 26787498

[17] AbuRahma AF, Hayes JD, Flaherty SK, Peery W. Primary iliac stenting versus transluminal angioplasty with selective stenting. J Vasc Surg 2007; 46:965–70. 10.1016/j.jvs.2007.07.027 17905559

[18] Yuan L, Bao J, Zhao Z, Feng X, Lu Q, Jing Z. Endovascular therapy for long-segment atherosclerotic aortoiliac occlusion. J Vasc Surg 2014; 59:663–8. 10.1016/j.jvs.2013.09.005 24239521

[19] Kim TH, Ko YG, Kim U, Kim JS, Choi D, Hong MK, et al Outcomes of endovascular treatment of chronic total occlusion of the infrarenal aorta. J Vasc Surg 2011; 53:1542–9. 10.1016/j.jvs.2011.02.015 21515016

[20] Ozkan U, Oguzkurt L, Tercan F. Technique, complication, and long-term outcome for endovascular treatment of iliac artery occlusion. Cardiovasc Interv Radiol 2010; 33:18–24. 10.1007/s00270-009-9691-7 19768500

[21] Müller A, Langwieser N, Bradaric C, Haller B, Fusaro M, Ott I, et al Endovascular treatment for steno-occlusive iliac artery disease: safety and long-term outcome. Angiology 2018; 69(4):308–15. 10.1177/0003319717720052 28747061

[22] Suzuki K, Mizutani Y, Soga Y, Iida O, Kawasaki D, Yamauchi Y, et al Efficacy and safety of endovascular therapy for aortoiliac TASC D lesions. Angiology 2017; 68(1):67–73. 10.1177/0003319716638005 26980775

[23] Ye W, Liu CW, Ricco JB, Mani K, Zeng R, Jiang J. Early and late outcomes of percutaneous treatment of TransAtlantic Inter-Society Consensus class C and D aorto-iliac lesions. J Vasc Surg 2011; 53(6):1728–1737. 10.1016/j.jvs.2011.02.005 21609804

[24] Soga Y, Lida O, Kawasaki D, Yamauchi Y, Suzuki K, Hirano K, et al Contemporary outcomes after endovascular treatment for aorto-iliac artery disease. Circ J 2012; 76:2697–704. 10.1253/circj.cj-12-0492 22864278

[25] Soares RA, Matielo MF, Brochado-Neto FC, Cury MVM, Costa VB, Sanjuan MCP, et al Factors associated with outcome of endovascular treatment of iliac occlusive disease: a single-center experience. J Vasc Bras 2018; 17(1):3–9. 10.1590/1677-5449.003817 29930675 PMC5990269

[26] Indes JE, Pfaff MJ, Farrokhyar F, Brown H, Hashim P, Cheung K, et al Clinical outcome of 5358 patients undergoing direct open bypass or endovascular treatment for aortoiliac occlusive disease: a systematic review and meta-analysis. J Endovasc Ther 2013; 20(4):443–55. 10.1583/13-4242.1 23914850

[27] Datillo PB, Tsai TT, Garcia JA, Allshouse A, Casserly MB, et al Clinical outcomes with contemporary endovascular therapy of iliac artery occlusive disease. Catheter Cardiovasc Interv 2012; 80:644–54. 10.1002/ccd.23469 22419505

[28] Papakostas JC, Chatzigakis PK, Peroulis M, Avgos S, Kouvelos G, Lazaris A, et al Endovascular treatment of chronic total occlusions of the iliac arteries: early and midterm results. Ann Vasc Surg 2015; 29:1508–15. 10.1016/j.avsg.2015.07.011 26315790

