# Supplementary material for: Endovascular treatment of different types of iliac occlusions—Results from an observational study
Source: PLoS One. 2019 Oct 2;14(10):e0222893. doi: 10.1371/journal.pone.0222893 (PMC6774573; doi:10.1371/journal.pone.0222893)
Supplement: S2 File — (DOC) [file pone.0222893.s002.doc]

"EARLY AND LONG-TERM RESULTS OF ENDOVASCULAR TREATMENT OF

AORTO-ILIAC OCCLUSIVE DISEASE"


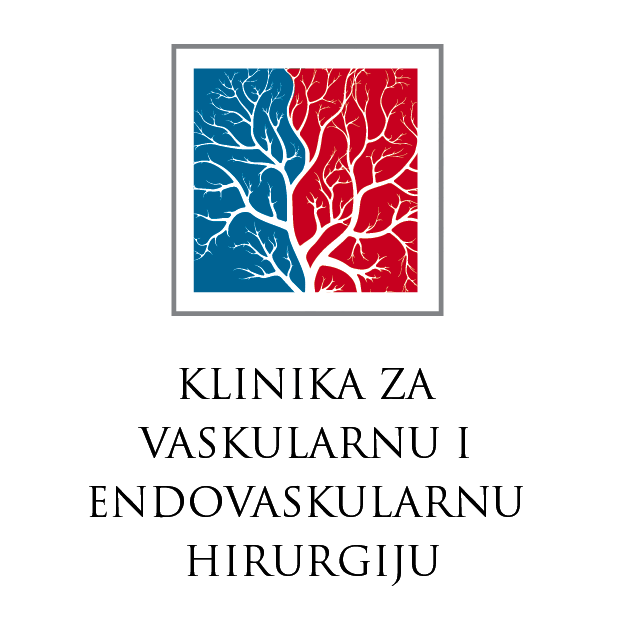

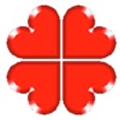


CLINIC FOR VASCULAR AND ENDOVASCULAR SURGERY

CLINICAL CENTER OF SERBIA

INSTITUTE FOR CARDIOVASCULAR DISEASES „DEDINJE“

**INFORMATION FOR PARTICIPANTS IN WRITTEN FORM**

You are being asked to participate in a medical research study. The aim of this study is to evaluate effects of endovascular treatment of aorto-iliac occlusive disease. Before agreeing to participate in this research study, it is important that you read the following explanation of this research study.

Please read this form carefully. Feel free to discuss the study with your family, friends, and healthcare provider before you make your decision whether to participate. Ask about anything you don’t understand or would like explained better. Take time to decide whether or not you want to take part in this study and ask the study doctor or study staff as many questions about the study as you would like. You cannot take part in this research study until you sign this form.

Thank You for your time!

***What is the purpose of this research, and why are you chosen?***

You are being asked to take part in this study because you have been diagnosed with aorto-iliac occlusive disease that requires endovascular treatment (according the international guidelines). The endovascular treatment is not a new therapeutic method, we would like to improve the treatment of aorto-iliac occlusive disease and we want to follow-up early and long-term effects of endovascular treatment in large number of patients, because we expect better early and long-term results, shorter recovery and less work capability.

***Are you obliged to participate?***

Your decision to help monitor the effects of endovascular treatment does not in any way affect your treatment nor are you required to participate in it, but we are obliged to inform you precisely about this.

***What will happen if you decide to participate in monitoring the effects of the endovascular therapy?***

Please only if you accept participation, you regularly come to the scheduled controls, and fill out the forms that allow us to get your opinion and attitude about the progress in treatment.

***What are the potential benefits and risks of participation?***

If we want to recognize the benefits of participation, they are certainly oriented towards a more accurate assessment of the effects of endovascular treatment, while the risks must not exceed the level of that the therapeutic approach otherwise carry with it.

***Will your participation and all data associated with it be kept as confidential information?***

All data that we collect by analyzing the effects of monitoring your illness, therapeutic approach are the level of medical secrets for which your medical doctor and full health staff are responsible.

***What is the purpose of this research and what will be the results?***

These data can be used exclusively as aggregated data for a larger number of patients who are involved in monitoring the effects of the endovascular treatment of aorto-iliac occlusive disease, and on the basis of a large number of results of this monitoring, we can, in all other cases of aorto-iliac occlusive disease, enable a more precise therapeutic approach than it was until now.

***Who organizes and finances this research?***

Additional funding for this research is not foreseen and is done exclusively within the framework of the usual diagnostic and therapy procedures in aorto-iliac occlusive disease.

***What will happen if you are worried, if you have any questions about this research, and / or if there is an emergency situation?***

You will be able to contact your doctor who is responsible for this research, to talk about what concerns you or to seek help: MD Vladimir Cvetić (Clinic for Vascular and Endovascular Surgery, Clinical Center of Serbia) and Prof. Dragan Sagic (Institute for Cardiovascular Diseases “Dedinje”).

If you accept to participate in this research, we will give you a copy of this information sheet and a signed form of informed consent.

"EARLY AND LONG-TERM RESULTS OF ENDOVASCULAR TREATMENT OF

AORTO-ILIAC OCCLUSIVE DISEASE"


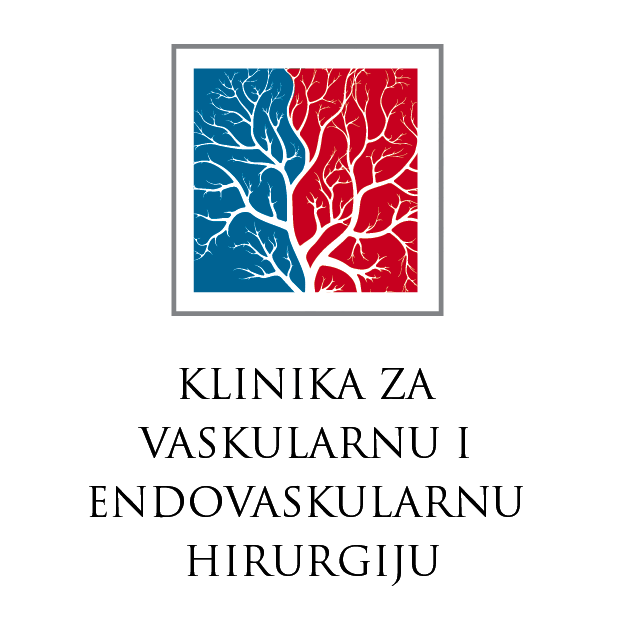

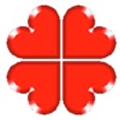


CLINIC FOR VASCULAR AND ENDOVASCULAR SURGERY

CLINICAL CENTER OF SERBIA

INSTITUTE FOR CARDIOVASCULAR DISEASES „DEDINJE“

**CONFIRMATION FOR PARTICIPATION IN RESEARCH**

**Name ________________________________________________**

**Participant number_________**

**I confirm that**

- I have read the foregoing information, or it has been read to me
- I have had the opportunity to ask questions about it and any questions that I have asked
- I understand that my participation is completely voluntary and I have right to withdraw from the study at any time without any implications to me
- I understand that only medical professionals, medical staff from Clinic for Vascular and Endovascular Surgery of Clinical Centre of Serbia and/or Institute for Cardiovascular Diseases Dedinje will have access to my medical record. All personal data will be treated as CONFIDENTIAL
- I am able to request a copy of the research findings and reports and that there is complete security and confidentiality of my personal information.
- I understand that all data collected during this study will be collected in the database and analysed only in scientific purpose
- I will receive a signed copy of this document
- I agree to participate in this study

Print Name of Physician: Print Name of Participant:

Date: Date:

Signature of Physician: Signature of Participant:
